# Microbiological Risks to Health Associated with the Release of Antibiotic-Resistant Bacteria and β-Lactam Antibiotics Through Hospital Wastewater

**DOI:** 10.3390/pathogens14050402

**Published:** 2025-04-23

**Authors:** Andres E. Nolasco-Rojas, Eder Cruz-Del-Agua, Clemente Cruz-Cruz, Miguel Ángel Loyola-Cruz, Benjamín A. Ayil-Gutiérrez, María C. Tamayo-Ordóñez, Yahaira de J. Tamayo-Ordoñez, Araceli Rojas-Bernabé, Francisco A. Tamayo-Ordoñez, Emilio M. Durán-Manuel, Marianela Paredes-Mendoza, Laura M. Márquez-Valdelamar, Carlos A. Jiménez-Zamarripa, Esther Ocharan-Hernández, Paola B. Zárate-Segura, Omar García-Hernández, Oscar Sosa-Hernández, Enzo Vásquez-Jiménez, Claudia C. Calzada-Mendoza, Juan M. Bello-López

**Affiliations:** 1 Hospital Juárez de México, Mexico City 07760, Mexico; 2 Sección de Estudios de Posgrado e Investigación, Escuela Superior de Medicina, Instituto Politécnico Nacional, Mexico City 11340, Mexico; 3 SECIHTI-Centro de Biotecnología Genómica, Instituto Politécnico Nacional, Biotecnología Vegetal, Reynosa 88710, Mexico; 4 Laboratorio de Ingeniería Genética, Departamento de Biotecnología, Facultad de Ciencias Químicas, Universidad Autónoma de Coahuila, Saltillo 25280, Mexico; 5 Laboratorio de Biotecnología Ambiental del Centro de Biotecnología Genómica, Instituto Politécnico Nacional, Reynosa 88710, Mexico; 6 Facultad de Química, Universidad Autónoma del Carmen, Ciudad del Carmen 24180, Mexico; 7 División de Tecnología Ambiental, Universidad Tecnológica de Nezahualcóyotl, Nezahualcóyotl 57000, Mexico; 8 Laboratorio de Secuenciación Genómica, LaNaBio, Instituto de Biología, UNAM, Mexico City 04510, Mexico; 9 Departamento de Dietología, Hospital Psiquiátrico “Dr. Samuel Ramírez Moreno”, Valle de Chalco Solidaridad 56619, Mexico; 10 División de Investigación, Facultad de Medicina, Universidad Nacional Autónoma de México, Mexico City 54090, Mexico

**Keywords:** hospital wastewater, ESKAPE bacteria, antimicrobial resistance, antibiotics

## Abstract

Hospital wastewater (HWW) is a major source of microbiological contamination, often released into the municipal sewage system without prior treatment. This HWW may contain pathogens with antimicrobial resistance, posing risks to public health. The aim of this work was to assess the risks associated with the seasonal release of ESKAPE bacteria resistant to β-lactam antibiotics and the release of carbapenems and cephalosporins through HWW of Hospital Juárez de México. The 12-month seasonal variation in β-lactam-resistant bacterial populations was assessed in the HWW of five discharge points. Resistant isolates were identified by mass spectrometry (MALDI-TOF) coupled with PCR assays to search for antimicrobial resistance genes, while β-lactamic antibiotics were detected using high-performance liquid chromatography (HPLC). Finally, a double-entry Vester matrix was constructed to classify the problems of HWW according to their degree of causality. Seasonal differences in bacterial loads were observed, with higher levels in warmer months. A wide variety of resistant pathogens were identified, including ESKAPE bacteria, as well as emerging bacteria carrying β-lactamase-encoding genes. The release of meropenem was detected most commonly, followed by cefepime and ceftazidime. The Vester matrix allowed the identification of critical clinical and environmental scenarios where two discharge points contribute significantly to the spread of microbiological contamination. This study highlights the importance of proper management of HWW and the need for stricter regulations to reduce the risks associated with the release of resistant pathogens with health impacts.

## 1. Introduction

Hospital wastewater (HWW) contains a wide range of chemical and microbiological contaminants as a result of the healthcare activities of various hospital services, including operating theatres, intensive care units, consulting rooms, diagnostic laboratories, medical device reprocessing areas, laundry, research units and laboratories, canteens, patient, and worker and visitor toilets, among others [1,2,3]. This is why, in recent years, research on HWW has gained great relevance, because it has been reported that this wastewater can be five to fifteen times more polluted than domestic wastewater, making it an important source of chemical and microbiological contamination of the environment, with potential impacts on public health [4]. This problem is increased in developing countries, where there is no legislation or regulations governing the pretreatment of HWW before its release into the municipal sewage system, so bacteria of nosocomial origin, mainly those of the ESKAPE group which are resistant to antibiotics represent a biological risk due to their ability to generate community infections and transfer mobile genetic elements, such as plasmids, transposons, and integrons that confer antimicrobial resistance, to aquatic microorganisms, such as diverse species of *Aeromonas* genus and others [5,6,7,8,9,10]. In this context, nosocomial bacteria (ESKAPE bacteria) have typically been considered as pathogens of healthcare-associated infections (HAIs), such as ventilator-associated pneumonia, catheter-associated bloodstream and urinary tract infection, and surgical site infection; however, although few studies exist, ESKAPE nosocomial pathogens have been recognized as having the capacity to cause community-acquired infections, meaning that they can cause infections in the general population, particularly in people with weakened immune systems when they come into contact through various routes, including HWW [11]. Examples of these pathogens include methicillin-resistant *Staphylococcus aureus* (MRSA), vancomycin-resistant *Enterococcus* spp., *Pseudomonas aeruginosa*, *Escherichia coli*, *Klebsiella pneumoniae*, *Citrobacter* spp., multidrug-resistant *Acinetobacter baumannii*, and, among others, *Enterobacterales* [12,13,14,15,16]. Nevertheless, local evidence has shown the microbiological risk associated with HWW, through classical microbiological culture isolation of a fraction of HWW on selective and differential culture media, followed by phenotypic and genetic characterization of antimicrobial resistance mechanisms in carbapenemase-producing *Enterobacterales* and other Gram-negative bacilli [17]. Nonetheless, weaknesses have been detected in these studies, as they do not allow a comprehensive assessment of the risks associated with HWW, as they only studied a small fraction of the total resistant bacterial diversity of medical interest present in HWW, and they do not show the impact of certain variables that could influence the biological risks of HWW, such as microbiological load, antibiotic-resistant subpopulations, seasonality, and the influence of environmental characteristics and contaminants, such as antibiotics. Another factor that adds to the problem of HWW is the corelease of antibiotics through it; this activity favors the selection of resistant strains of medical interest, emerging bacteria, and bacteria native to the aquatic environment, which increases the risk of vulnerable individuals acquiring infections that are difficult to treat with current antimicrobial therapies [5]. Therefore, the aim of this study was to assess the risks associated with the release of antibiotic-resistant nosocomial bacteria (ESKAPE group) through the hospital wastewater of the Hospital Juárez de México (HJM), a tertiary hospital located north of Mexico City. This was carried out through a temporal study of a joint evaluation of the parameters of total microbiological load, resistant populations, taxonomic diversity, circulating antimicrobial resistance mechanisms, seasonality, and contamination by antibiotics (β-lactamics) used in the treatment of hospital-acquired infections (cephalosporins and carbapenems). The risks associated with the release of ESKAPE bacteria, emerging pathogens resistant to cephalosporins and carbapenems in HWW, are analyzed and discussed.

## 2. Materials and Methods

### 2.1. HWW Discharge Points of the HJM

**Figure 1:**
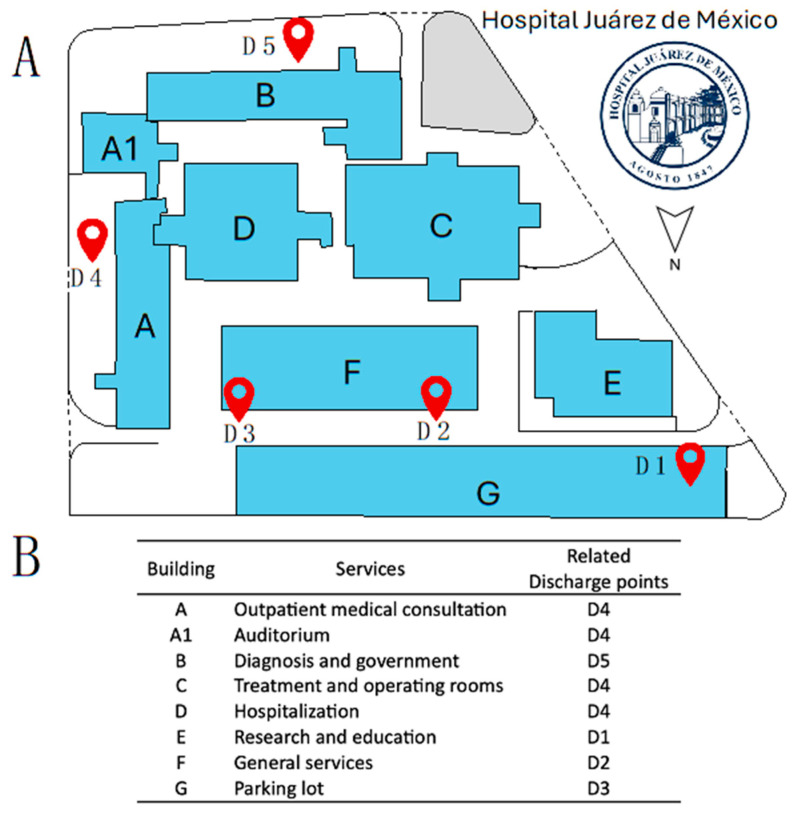
(**A**) Aerial map of the Hospital Juárez de México with the discharge points (D1 to D5) of HWW, and (**B**) hospital services related to each of the discharge sites. The discharge points (D1 to D5) are indicated by the red marking symbol.

The Hospital Juárez de Mexico (HJM) has five HWW discharge points that discharge directly into the municipal public sewage system of Mexico City without prior treatment (D1 to D5) (Figure 1). These discharge points are characterized as being sites where HWW from various sites in the hospital converges, including critical areas that release significant loads of microbiological contamination, such as intensive care units for adult, pediatric, and neonatal patients, certain services, such as endoscopy, reprocessing areas for invasive medical devices (endoscopes, mechanical ventilators, surgical equipment, etc.), diagnostic laboratories, and research laboratories, among others. The HWW from these discharge points was analyzed physiochemically and microbiologically for one year. Figure 1 shows the aerial map of the HJM with the HWW discharge points (D1 to D5) and the hospital services related to each HWW discharge point.

### 2.2. HWW Collection and In Situ Temperature Determination

This study was observational, descriptive, and cross-sectional, and took place from January to December 2024, distributed over 12 monthly monitoring sessions (one per month/per discharge point) with a total of 60 HWW samples. For the microbiological and quality analysis of HWW from each discharge point (D1 to D5), 200 mL of HWW was collected in sterile amber glass bottles according to the Mexican Standard NMX-AA-003-1980 “that establishes the characteristics of sampling of residual waters” [18]. The following aspects were considered for the standardized taking of HWW: the 15th of each month, and a fixed timetable of 10:00 a.m., as this is the busiest time in the hospital. In situ temperature determination was performed at the time of HWW sample collection in accordance with the Mexican standard NMX-AA-007-SCFI-2000 “Determination of temperature in natural, waste and treated wastewater” [19]. HWW samples were collected in strict adherence to biosafety conditions and transported at 4 °C to the microbiology laboratory, ensuring processing within a maximum of 20 min after collection (without preserving agents).

### 2.3. Determination of HWW Quality Standard Parameters

For the determination of HWW quality standard parameters, standardized methodologies were used based on the Mexican Standards for domestic and treated wastewater, i.e., NMX-AA-108-SCFI-2001 and NMX-AA-008-SCFI, for the determination of residual chlorine and pH, respectively [20,21]. Significant differences in temperature, residual chlorine, and pH between discharge points were analyzed and evaluated in the study period using ANOVA and Tukey’s post hoc. Significant differences were established when the *p*-value was <0.05. Additionally, the SPSS v.27.0.1.0 and XLSTAT 2023 statistical software programs were used for analysis and graphical representation.

### 2.4. Quantification of β-Lactam-Resistant Bacterial Populations in HWW

For the quantification of β-lactam-resistant Gram-negative aerobic mesophilic populations, we considered the antibiotics that, according to the institutional Antimicrobial Steering Committee, were the most frequently used in antimicrobial therapy during 2024 (cephalosporins and carbapenems) in the treatment of nosocomial infections in HJM. For this purpose, a classical microbiological culture protocol based on the seeding of appropriate serial dilutions onto selective MacConkey agar plates containing ceftazidime (8 μg/mL), cefepime (16 μg/mL), and meropenem (8 μg/mL) was employed. In parallel, total aerobic mesophilic bacteria (on trypticase soy agar (TSA) without antibiotics) and total Gram-negative bacteria (on MacConkey agar without antibiotics) were quantified (per triplicate). The plates were incubated at 35 ± 2 °C for 24 and 48 h and were counted and reported as colony forming units per milliliter of HWW (CFU/mL) [22,23]. Antibiotic concentrations were based on the clinical cut-off points recommended by the Clinical and Laboratory Standards Institute for the classification of Enterobacteriaceae as “resistant” (CLSI 2024) [24]. With the information obtained, an analysis using ANOVA and Tukey’s post hoc was performed to identify significant differences in the behavior of bacterial loads per discharge point (D1–D5) in the study period (*p* < 0.05). Finally, relative abundance heat maps were constructed to determine the seasonal behavior of each resistant population, and a Spearman correlation analysis (non-parametric) was performed to determine the correlations between microbiological loads and standard parameters (temperature, residual chlorine, and pH). Colonial resistant morphotypes were selected per antibiotic during the study period (*n* = 155) and tested for antimicrobial resistance with the same antibiotics as primary isolates using the disc diffusion test according to the CLSI guidelines. They were then purified onto TSA agar for identification at the genus and species levels, as follows.

### 2.5. Bacterial Identification by Mass Spectrometry (MALDI-TOF)

Resistant bacterial isolates (*n* = 155) were identified at the genus and species levels via the direct analysis of whole bacterial cells using matrix-assisted laser desorption/ionization–time-of-flight mass spectrometry (MALDI-TOF MS). For this purpose, all strains were streaked in LB agar and incubated overnight at 37 °C, and single colonies were subjected to identification by using a Bruker MALDI Biotyper (Bruker Daltonik, Germany) according to the manufacturer’s instructions. The criteria to best match with the identification protocol were bacterial strains with score values above 2.0 (down to 3) for high-confidence identification. MBT Compass Library version 10.0 was used to assign genus and species to the isolates. Finally, an alluvial diagram was generated in RAWGraph for antimicrobial resistant bacteria liberation (ESKAPE, emergent, and others) by discharge points (D1 to D5) [25].

### 2.6. Detection of β-Lactam Resistance Genes in Resistant Bacteria

In accordance with the local epidemiology of circulating antimicrobial β-lactam resistance mechanisms in ESKAPE pathogens from the HJM, isolates were subjected to total DNA extraction and subsequently to endpoint PCR assays to detect three metallo-β-lactamase (*bla_NDM_*, *bla_VIM_*, and *bla_IMP_*) and four serine β-lactamase (*bla_KPC_*, *bla_OXA-48_*, *bla_OXA-23_*, and *bla_OXA-40_*) genes, using the primers according to Cureño-Díaz et al., 2024 [26]. Genomic DNA was extracted using the QIAamp DNA Mini QIAcube Kit (QIAGEN, Hilden, Germany) according to the instructions provided by the manufacturers. The reactions were performed in a Touchgene Gradient thermal cycler FTGRAD2D (TECHNE DUXFORT, Cambridge, UK) using MasterMix PCR 1× (Roche Diagnostics, Hilden, Germany), with 200 pmol of each primer and 200 ng of template DNA. Amplicons were run and separated via electrophoresis, visualized, and photographed under UV illumination. Appropriate controls (negative and positive) were obtained from Cureño-Díaz et al. (2024), Cortés-Ortíz et al. (2021), and Loyola-Cruz et al. (2023) [26,27,28]. With the genetic information of antimicrobial resistance and origin of detection, a chord plot analysis was performed to know the distribution of resistance genes by ESKAPE bacteria, emerging pathogens and those classified as “other” during the study period [29].

### 2.7. Detection and Quantification of β-Lactam Antibiotics in HWW

High-performance liquid chromatography (HPLC) was used for the detection and quantification of β-lactam antibiotics in HWW (meropenem, ceftazidime, and cefepime). An HPLC 1100 chromatographic system consisting of a quaternary pump (Agilent Technologies G1310A, Waldbronn, Germany) connected to an automated injector (Agilent Technologies G1314A, Hachioji, Tokyo, Japan), with a C18 reverse-phase column (Eclipse Plus C18, 4.6 × 150 mm 5-Micron Agilent) was used. The antibiotics meropenem, ceftazidime, and cefepime were detected at a wavelength of 296 nm with a system flow rate of 0.350 mL/min at 25 °C, with a 70:30 MeOH/H_2_O run system. The limit of detection (LOD) for each of the antibiotics, i.e., meropenem, ceftazidime and cefepime was 4.99 × 10^−5^, 6.938 × 10^−7^, 6.4 × 10^−8^ mg, respectively, and the limit of quantification (LOQ) was 0.0002, 4 × 10^−6^, 1 × 10^−7^ mg, respectively. HPLC-grade antibiotics were used for the construction of calibration curves (mg/mL). Significant differences in antibiotics between discharge points were analyzed and evaluated using ANOVA and Tukey’s post hoc (*p* < 0.05).

### 2.8. Identification of Risks Associated with HWW by Means of a Vester Matrix

Using the microbiological and physicochemical findings, a Vester matrix was constructed to classify the HJM HWW situations according to their degree of causality. For this purpose, 30 situations were identified that could have an impact on various health risk outcomes. The 30 situations with potential health impacts were identified through consensus among a group of professionals in the fields of infectious disease, infection control, microbiology, and environmental engineering, along with a review of the scientific literature. Causality was assessed individually by a second group of various professionals, including 5 microbiologists, 5 environmental engineers, 5 ecologists, 5 medical infectiologists, and 5 epidemiologists. With the causality results (0, 1, 2, and 3), those that occurred most frequently were considered to have the closest causality to reality and were transferred to the matrix to categorize them as active, passive, critical, or indifferent problems [30].

## 3. Results

### 3.1. Evaluation of Standard HWW Parameters

**Figure 2:**
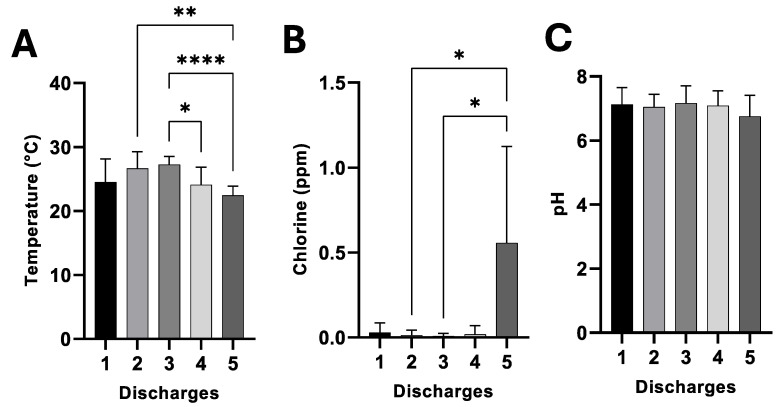
ANOVA and Tukey’s post hoc of standard parameters of HWW at the discharge points (D1 to D5) of the HJM during 2024. (*p* < 0.05) (**A**) Temperature, (**B**) residual chlorine, and (**C**) pH. Asterisks represent varying levels of significance.

Figure 2A–C shows the results of the three standard parameters of the HWW at the five discharge points (D1 to D5) of the HJM during 2024. The ANOVA and Tukey’s post hoc analysis demonstrated significant differences with varying degrees of statistical significance between the five discharge points for two (temperature and chlorine residual) of the three parameters determined (Figure 2A,B). Temperature and residual chlorine showed varying levels of significance between discharge points (*p* = 0.0001 and 0.00001) compared to pH, which showed no significant difference and was classified as neutral to slightly alkaline for HWW (Figure 2C). Discharge points two and three stand out as releasing HWW with the highest temperatures, with values of 27.4 ± 2.17 and 27.5 ± 1.32 °C, respectively, while discharge point five stands out as actively releasing large amounts of residual chlorine (0.65 ± 0.63 ppm).

### 3.2. Quantification of Bacterial Populations in HWW

**Figure 3:**
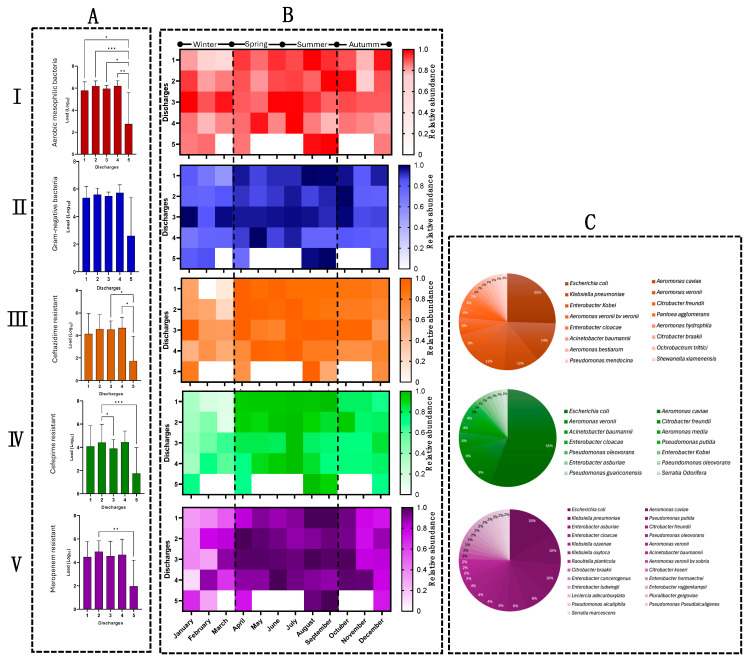
Quantification and identification of bacteria released through HWW at the discharge points (D1 to D5) of the HJM. (**A**) ANOVA and Tukey’s post hoc analysis of total mesophilic and β-lactam-resistant loads (*p* < 0.05); (**B**) seasonal heat maps of total mesophilic and β-lactam-resistant loads; (**C**) β-lactam-resistant isolates by MALDI-TOF mass spectrometry. I. Aerobic mesophilic bacteria, II. Gram-negative bacteria, III. Ceftazidime resistant, IV. Cefepime resistant, and V. Meropenem resistant. Asterisks represent varying levels of significance.

Figure 3 summarizes the results of the quantification and identification of the bacterial populations released through the HWW during one year at the five discharge points of the HJM. As can be observed, the aerobic mesophilic microbiological load released through the HWW was at an average magnitude of 5.3 ± 1.4 Log_10_. Discharge point 5 (D5) was where the lowest microbiological load was released (2.74 Log_10_) and, coincidentally, it was also the point where the highest levels of residual chlorine were identified (Figure 2B and Figure 3(AI)). Conversely, the differential quantification of populations showed that the Gram-negative population represented close to the total aerobic mesophilic population in the discharges during the whole study period, with minimum and maximum values of 92.3 to 94.8% for discharge points D3 and D5, respectively, even though the ANOVA test showed that the five discharge points released similar loads of this subpopulation (2.60 to 5.58 Log_10_) (Figure 3(AII)). Regarding the quantification of the β-lactam-resistant subpopulations, the results revealed that discharge point 5 still stood out as releasing the lowest Gram-negative resistant microbiological load, with values of 1.7, 1.75, and 1.95 Log_10,_ for the antimicrobials ceftazidime, cefepime, and meropenem, respectively. In contrast, the remaining discharges showed high levels of microbiological loads resistant to the three antibiotics, with maximum loads of 4.7, 4.4, and 4.9 Log_10_, for both cephalosporins at discharge point 4 (D4) and carbapenem at discharge point 2 (D2), respectively (Figure 3(AIII–AV)). Finally, the ANOVA test showed at least one significant difference (meropenem) in the release of resistant bacteria per discharge point, with discharge points 2 and 4 standing out as the points where the highest resistant microbiological loads were released.

### 3.3. The Release of β-Lactam-Resistant Bacteria Shows Seasonal Behavior

Seasonal analysis using heat maps of relative abundance of bacterial load showed that the warm seasons (spring and summer) were those where the highest resistant microbiological load was released (Figure 3(BI–BV)). However, even though the temperature of these seasons possibly influenced the concentration of the bacterial load released, discharge points, such as D3 and D4 showed active release of significant concentrations of resistant and non-resistant bacteria throughout the year. The presence of significant concentrations of residual chlorine at discharge point 5 directly impacted the recovery of resistant bacteria in seven of the twelve months (spring and summer), even when subjected to concentration by centrifugation. As mentioned, we speculated that seasonal environmental temperatures influenced the release of different loads of total and antibiotic-resistant bacteria. To test this hypothesis, a Spearman analysis (non-parametric) was performed to determine if there was a correlation between seasonal microbiological loads (total Gram-negative bacteria) and environmental temperature. The results showed a positive correlation between the variables (*Rho* = 0.771, *p* = 0.01, CI 95% = 0.337 to 0.935), indicating a dependence between the quantified microbiological load and the environmental temperature. This analysis was performed with the other variables studied (residual chlorine and pH), showing the presence of a negative correlation for the residual chlorine parameter *(Rho* = −0.662, *p* = 0.02, CI 95% = −0.899 to −0.122), indicating that the bactericidal activity of chlorine directly influences the detectable bacterial load even when environmental temperature influences bacterial growth. Finally, no correlation was identified between pH (classified as neutral to slightly alkaline HWW) and detectable microbiological loads (*Rho* = 0.787, *p* = 0.088, CI 95% = −0.526 to −0.641).

### 3.4. Microbiological Identification Revealed ESKAPE Bacteria and Emerging Pathogens in HWW

**Figure 4:**
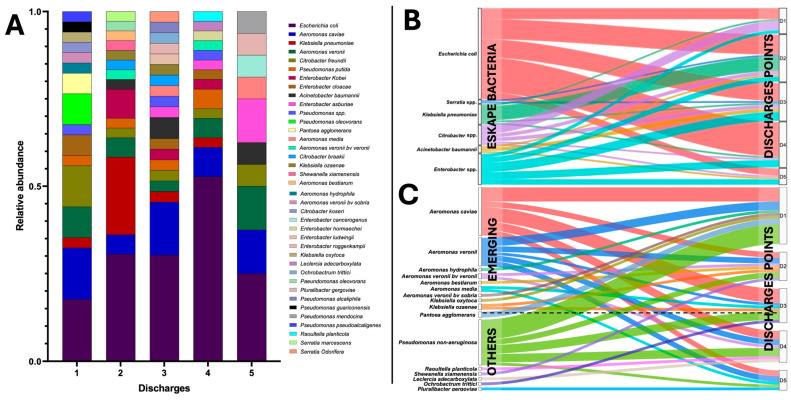
Taxonomic diversity of β-lactam-resistant bacteria isolated by discharge point from HWW of the HJM. (**A**) Relative abundance of the taxonomic diversity by HWW discharge point, (**B**) alluvial analysis by HWW discharge point, classified by ESKAPE bacteria (marked by colors for each bacteria), and (**C**) emerging and “other” bacteria (marked by colors for each bacteria). The dotted line indicates the separation of ESKAPE and emerging bacteria.

Analysis of the diversity of the 155 isolates obtained from HWW revealed 16, 14, and 25 genera and species resistant to ceftazidime, cefepime, and meropenem, respectively, and included ESKAPE bacteria, such as *E. coli*, *K. pneumoniae*, *A. baumannii*, *Serratia* spp., *Citrobacter*, and *Enterobacter* (Figure 3C). *E. coli* was the predominant ESKAPE bacteria, with frequencies of 16%, 25%, and 55% for meropenem, ceftazidime, and cefepime, respectively. The ESKAPE members with the lowest frequency after *E. coli* were represented by *K. pneumoniae*, *Enterobacter* spp., *A. baumannii*, and *Serratia* spp. A relevant finding was the identification of bacteria resistant to at least one β-lactam and of emerging clinical relevance, such as *K. oxytoca*, *K. ozaenae*, *Aeromonas veronii*, *A. bestiarum*, *A. caviae*, *A. hydrophila*, and *A. media*. The presence of non-ESKAPE and non-emerging bacterial genera categorized as “other”, such as *P. mendiocina*, *P. guariconensis*, *P. olevorans*, *Pantoea agglomerans*, *P. pseudialcaligenes*, *Ochrobactrum trittici*, *Shewanella xiamenensis*, *Paeundomonas olevorans*, and *Puribacter gergoviae*, was highlighted. Relative abundance analysis of genus and species data showed that the three resistant populations were grouped by HWW discharge point, showed 16, 15, 19, 19, 14, and 10 different genera and species for points D1, D2, D3, D4, and D5, respectively, with the *Enterobacterales* and *Aeromonas* species being predominant (Figure 4A). Interestingly, discharge points 3 and 4 stand out as active release points for fecal contamination due to the high relative abundance of *E. coli* and β-lactam-resistant *Enterobacter* species. The alluvial analysis of genera and species showed similar behavior in the distribution of ESKAPE bacteria across the discharge points (Figure 4B); however, it showed that the seven species of *Aeromonas* presented a similar distribution of abundance at the discharge points compared to *E. coli*, with *A. veronii* standing out as the predominant species (Figure 4C). Finally, a group of resistant bacteria classified as “other” was identified, where the group of *P.* non-*aeruginosa* was homogeneously distributed per discharge, in contrast to genera and species that were identified at a lower frequency and that were neither ESKAPE nor emerging but were resistant to the test antibiotics

### 3.5. Detection of Carbapenems and Cephalosporin Resistance Genes

**Figure 5:**
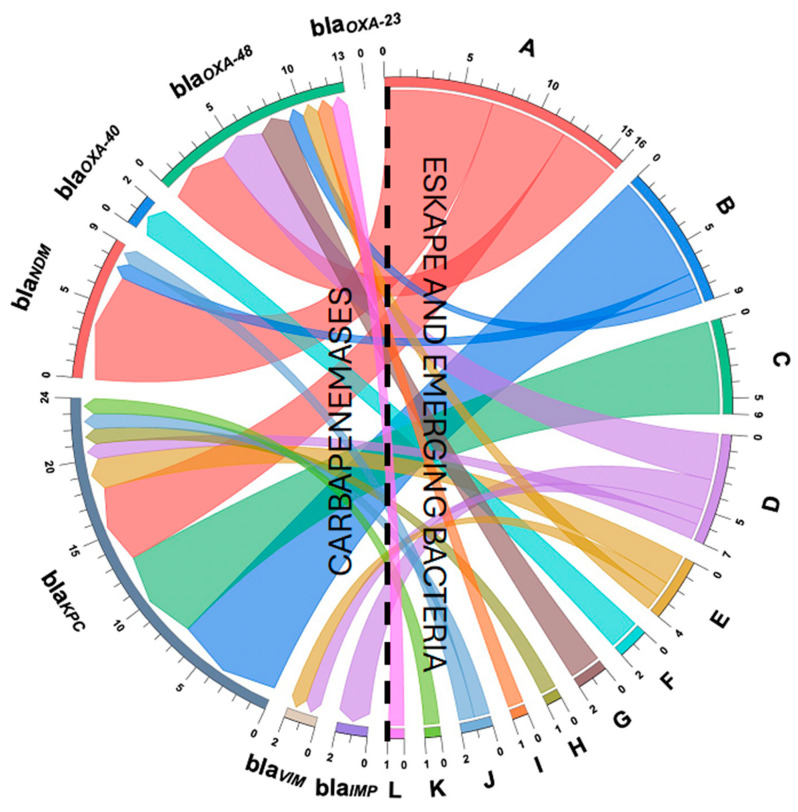
Chord plot of the distribution of β-lactam resistance genes of local epidemiological importance in ESKAPE and emerging pathogens and “other” strains isolated from HWW of the HJM. (**A**) *E. coli*; (**B**) *A. caviae*; (**C**) *C. freundii*; (**D**) *P.* non-*aeruginosa*; (**E**) *Enterobacter* spp.; (**F**) *A. baumannii*; (**G**) *K. pneumoniae*; (**H**) *A. media*; (**I**) *A. hydrophila*; (**J**) *K. oxytoca*; (**K**) *S. marcescens*; (**L**) *P. agglomerans*. The dotted line indicates the separation of antimicrobial resistance genes and ESKAPE /emerging bacteria.

The detection of β-lactamase resistance genes in carbapenem-resistant and cephalosporin-resistant isolates showed that, of the 155 isolates, only 44 (28.4%) had at least one antimicrobial resistance gene marker of local epidemiological significance. In contrast, for those isolates (*n* = 111) where high frequencies of resistance to the three antibiotics tested were identified, no molecular fingerprints of antimicrobial resistance were identified (Figure 5).

The genes coding for the serine β-lactamases *bla_KPC_* and *bla_OXA-48_* were most frequent in *A. caviae* (emerging pathogen), followed by ESKAPE bacteria, such as *C. freundii* and *E. coli*, in contrast to *bla_IMP_*, *bla_VIM_*, and *bla_OXA-40_*, which were less frequent in the genus “*P.* non-*aeruginosa”* and *A. baumannii*, respectively. Although the gene coding for the serine β-lactamases *bla_OXA-40_* and *bla_OXA-23_* is specific to the genus and species *A. baumannii*, all resistant isolates were screened with the absence of this gene. Finally, the analysis of the coexistence of resistance markers revealed that enterobacteria from the ESKAPE group, i.e., *E. coli*, *Enterobacter*, *K. pneumoniae*, and *P.* non-*aeruginosa*, showed the coexistence of serine and metallo β-lactamases, represented by the combination *bla_KPC_ + bla_NDM_* and *bla_VIM_* + *bla_OXA-48_* (Table 1). In the case of the emerging pathogen *A. caviae*, the coexistence of *bla_KPC_ + bla_NDM_* markers was identified. Figure 5 shows a chord plot analysis of the distribution of resistance markers of local epidemiological significance in ESKAPE and emerging β-lactam-resistant pathogens isolated from HWW from the HJM, and Table 1 shows the frequency analysis of the detected resistance genes of local epidemiological significance.

### 3.6. Release of β-Lactam Antibiotics Through HWW Discharges from the HJM

**Figure 6:**
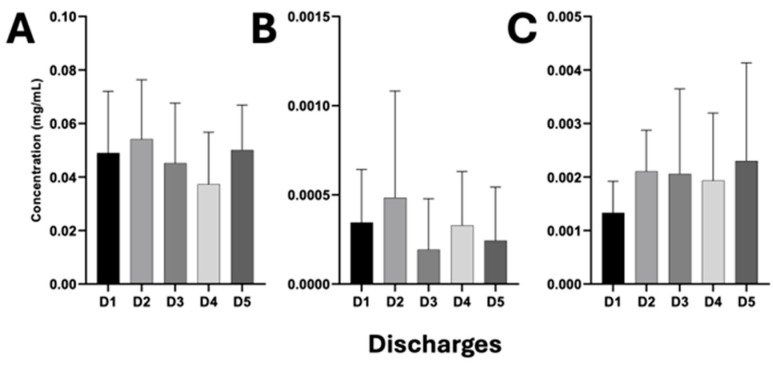
ANOVA and Tukey’s post hoc (*p* < 0.05) of the quantification of β-lactam antibiotics by HPLC of the HWW of the HJM. (**A**) Meropenem; (**B**) cefepime, and (**C**) ceftazidime.

Figure 6 shows the ANOVA analysis of the HPLC quantification of β-lactam antibiotics released through the HWW discharge points of the HJM. As observed in Figure 6A, the carbapenem antibiotic (meropenem) was detected in the highest concentration during the study period compared to the two cephalosporins analyzed (cefepime and ceftazidime), with average maximum values at discharge point 2, with values of 0.056 ± 0.02 mg/mL, and average minimum of 0.037 ± 0.02 mg/mL for discharge point 4. ANOVA analysis revealed the absence of significant differences in the detected concentrations of this antibiotic. The same case was observed for the cephalosporins analyzed, i.e., even though maximum average concentrations of these two antibiotics were identified for discharge point 2 (0.0045 mg/mL) and 5 (0.0023 mg/mL) for cefepime and ceftazidime, respectively, no significant differences were identified during the study period (Figure 6B,C).

### 3.7. Vester Matrix to Identify Problems Associated with HWW

**Figure 7:**
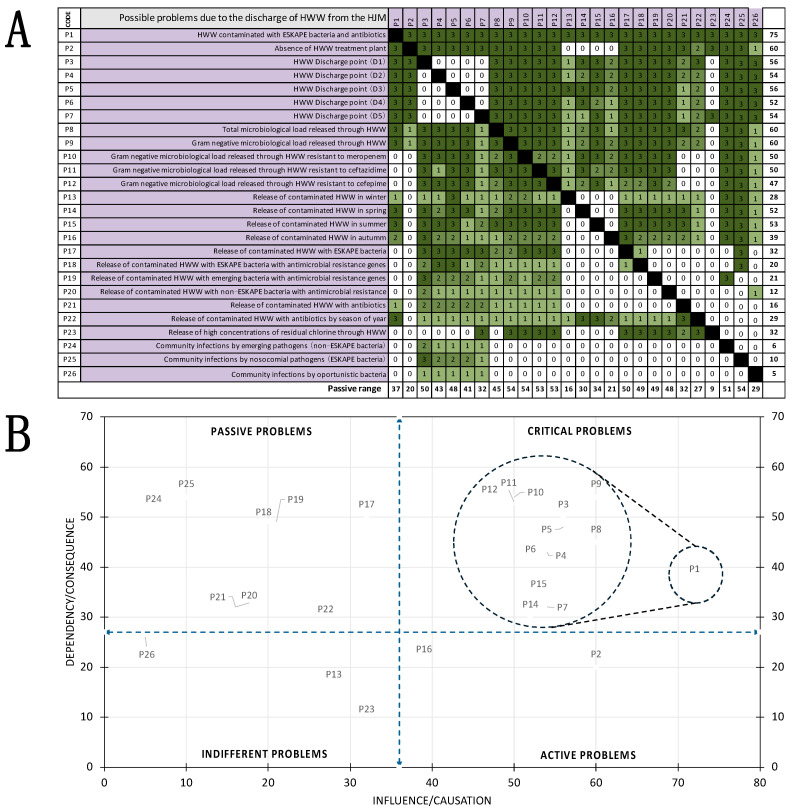
(**A**) Vester matrix constructed with twenty-six critical clinical and environmental scenarios due to HWW release from the HJM. (**B**) Spatial distribution of the 26 problems into passive, critical, indifferent, and active categories. The dotted lines indicate the classification and grouping of problems on the Cartesian plane.

With the findings from the microbiological, genetic, and physicochemical determinations, a Vester matrix was constructed to classify different situations or scenarios according to their degree of causality to determine which ones resulted in microbiological problems associated with HWW at the HJM. Twenty-six main critical clinical and environmental scenarios were identified (Figure 7A) where environmental variables, such as seasonality, HWW discharge points, bacterial subpopulation load, and others, were included.

Additionally, the degree of causality of the microbiological findings was analyzed according to the type of pathogen, i.e., ESKAPE, non-ESKAPE, and emerging pathogens, as well as the presence of carbapenems and cephalosporin resistance genes, chlorine, and antibiotic contamination. The results revealed that, of the critical clinical and environmental scenarios counted as “problems” (26/100%), 13 critical problems (50%), 8 passive problems (30.8%), 2 active problems (7.7%), and 3 indifferent problems (11.5%) were identified, with the release of HWW contaminated with bacteria and antibiotics being the predominant driver risk, from which the rest of the critical problems (P1s) are derived. This crucial finding highlights the urgent need to address the unregulated discharge of microbiologically and chemically contaminated HWW, which acts as a potential risk to environmental and public health. Particularly concerning is the absence of a HWW treatment plant (P9) and the release of large total microbiological loads and Gram-negative subpopulations (P8 and P9), which were both categorized as active problems. These scenarios not only demonstrate the risk of antimicrobial resistance propagation and environmental contamination but also show the lack of HWW treatment plants. Community infections by opportunistic bacteria, the release of contaminated HWW in winter, and elevated chlorine residual concentrations (P13, P23, and P26) were grouped as indifferent problems; however, they are closely related to critical problems if not mitigated (Figure 7B). Figure 7A shows the Vester matrix that was constructed with the 26 critical clinical and environmental scenarios for HWW release from the HJM. In addition, Figure 7B shows the spatial distribution of passive, critical, indifferent, and active problems.

## 4. Discussion

In recent years, HWW has gained interest as it has been recognized as a potential source of microbiological and chemical contamination released into the environment with impacts on human health. This problem is aggravated in those countries that do not have regulations limiting the release of HWW without prior treatment before discharge into the municipal sewage system. This is why we consider the information obtained from this study to be of importance, as it provides data on the problems associated with β-lactam antibiotic-resistant bacteria of the ESKAPE group with the potential to generate community infections and the presence of antibiotics in untreated HWW. In previous research by our working group, we have identified a close relationship between these microorganisms with difficult-to-treat HAIs in vulnerable patients; however, we have speculated about the negative impact of HWW due to hospital activities, with community-acquired infections being one of them [28,31,32]. Although our microbiological findings are in line with other previously published Mexican studies [17,33,34], they highlight public health issues associated with variables that to our knowledge had not been explored, such as resistant microbiological load (including ESKAPE bacteria) and its relationship with seasonality, as well as the presence of antibiotics in HWW.

The seasonal variability in bacterial load released in this study provides valuable information for understanding the impact of environmental factors, particularly temperature, as modulators in the release of antibiotic-resistant pathogenic bacterial loads (Figure 3B). As expected, high bacterial loads were detected during the warmer months (spring and summer), confirming that environmental temperature plays an important role in the growth of bacteria in hospital wastewater. Nevertheless, the resistant bacterial load that was released during the colder months highlights the permanent year-round risk posed by the discharge of untreated HWW. In addition, in terms of the results related to resistant microbiological loads, significant concentrations of antibiotic resistant bacteria were observed, which underlines the importance of focusing on efforts to mitigate the release of these microorganisms, especially at those discharge points associated with higher resistant bacterial loads, e.g., points D2 and D4, which are of particular concern and which were classified in the Vester matrix as critical problems (P4 and P6) (Figure 7). Risk assessment using such matrices allows for the identification of those problems that are of urgent attention, as by addressing them, these can be turned into active problems.

In this context, the mitigation of the release of resistant bacteria in HWW has already been addressed in some hospitals in our country, with treatment plants being one of the alternatives that are used for effectively reducing the prevalence of resistant pathogens [1]; however, these systems do not guarantee the total elimination of resistant microorganisms, since, even when these HWW treatment plants have state-of-the-art technology, the persistence of bacteria from the ESKAPE group has been demonstrated after wastewater treatment processes [35]. In this regard, Figure 3 shows that the bacterial loads (total and resistant) released through HWW are not only directly related to temperature, but also stand out for containing a wide variety of resistant pathogens, including ESKAPE group bacteria, such as *E. coli*, *K. pneumoniae*, *A. baumannii*, *P. aeruginosa*, *Enterobacter*, and *Citrobacter*, while at discharge point 5, free chlorine was a limiting factor for bacterial growth regardless of season (Figure 2B and Figure 3A,B). The isolation of β-lactam-resistant bacteria in HWW in the presence of large amounts of free chlorine (up to five times above the maximum permissible limits), in addition to being considered as a limiting factor for proliferation, could be a promoter of the persistence of resistant bacteria, as well as of the transfer of antibiotic resistance genes.

In a previous study, chlorine present in HWW was shown to promote the survival of extended-spectrum β-lactamase (ESBL)-producing bacteria and the transmission of genetic determinants of antimicrobial resistance. This work discusses how disinfection practices may represent a risk to the environment and public health beyond being a barrier to limiting bacterial growth [36]. Therefore, future work will be directed towards understanding the phenotype and genotype in terms of antimicrobial resistance in the population of bacteria that were isolated at discharge point 5 (Figure 3B,C). Interestingly, the genus *Aeromonas* also stood out as a prevalent bacterial group in the HWW along with the fecal contamination indicator “*E. coli*” at all the discharge points of the HJM (Figure 4A,C). The genus *Aeromonas*, in addition to being ubiquitous, is considered an emerging pathogen that has been reported as a potential generator of infections in humans and hospitalized patients [37,38,39,40]. The multidrug resistance of this bacterial genus has relied on its ability to donate and receive mobile genetic elements as resistance plasmids under in vitro, in vivo, and adverse temperature conditions [5,7]. The identification of this bacterial genus with antimicrobial resistance profiles highlights the importance of strengthening microbiological surveillance through the intentional search for this type of microorganism as a causative agent of nosocomial infections, since cultures are traditionally directed at the intentional search for bacteria from the ESKAPE group. Due to the abovementioned discussion, our current research focuses on the characterization of protein-coding genes that confer carbapenem and cephalosporin resistance in *Aeromonas* spp. and *A. baumannii*, including the differentiation between intrinsic and acquired resistance mechanisms. Experiments involving whole-genome sequencing and transcriptome analysis under selective pressure (carbapenems and cephalosporins) are underway to understand the resistomes and mobilomes of these microorganisms. These studies will allow a more in-depth evaluation of their relevance to bacteria released through HWW. The detection of several resistant *Aeromonas* species and genes related to mobile genetic elements (*bla_KPC_*), shows the concern of these species being considered environmental reservoirs of antimicrobial resistance to β-lactam antibiotics. Undoubtedly the identification of resistant bacteria that were not from the ESKAPE group, grouped in “others” (Figure 4C), should also be a cause for attention because of their ability to generate opportunistic infections in patients with comorbidities and the immunologically compromised [41,42,43,44,45,46].

It is important to consider that the presence of intrinsic resistance mechanisms to β-lactam antibiotics in certain Gram-negative bacterial species was not excluded during the quantification of resistant populations. Some isolates could present natural resistance, independent of acquired genes, as is the case with the *Aeromonas* genus, which possesses a constitutive chromosomal cephalosporinase [47]. Although confirmatory methods for resistance were performed at the phenotypic and genetic levels, additional evidence is required to distinguish between intrinsic and acquired resistance (due to mobile genetic elements). Now, in terms of antimicrobial resistance, techniques, such as massive sequencing, are widely used to characterize the resistome of HWW [35,48]. Our experimental approach allowed us to analyze HWW from a local epidemiological perspective (due to the resistance mechanisms circulating in our hospital) in order to specifically determine the resistance genes that were in the bacteria released through HWW. Therefore, we consider it important to identify antimicrobial resistance genes of local epidemiological interest in ESKAPE pathogens, emerging pathogens, and other isolates from this source (Figure 5 and Table 1).

Our findings are consistent with previous studies by our working group on the presence of *bla_VIM_*, *bla_NDM_*, *bla_OXA-40_*, *bla_OXA-48_*, *and bla_KPC_* genes in in-hospital pathogens, so we speculate that the presence of these detected resistance genes could be further evidence of the interconnection between the hospital setting and HWW. Further studies, such as clonality analysis by molecular methods or whole-genome sequencing, would serve to test this hypothesis, as this has been demonstrated in previously published work [10]. Conversely, the coexistence of these resistance genes further complicates the antimicrobial resistance landscape, as it is clear evidence of the presence of horizontal transfer events of genetic material that translates into limited treatment options for infections caused by these bacteria in community infections. Therefore, the phenomenon of gene transfer and spread through HWW demands an approach with a direct impact on hospital infection control. We believe that while the implementation of wastewater treatment plants may be far off, alternatives, such as the management of hazardous biological infectious waste (HBIW), exist to mitigate the release of nosocomial microbiological contamination. The release of antibiotics into the environment via HWW is considered a selection pressure factor for resistant strains, so the detection of antibiotics shows that the HJM is an active releaser into the environment, which undoubtedly contributes to the emergence and spread of resistant strains. As can be seen in Figure 6, the quantified antibiotic concentrations were at maximum magnitudes of 56, 4.5, and 2.3 µg/L, for meropenem, cefepime and ceftazidime, respectively. These concentrations are higher than the predicted no effect concentrations (PNEC) for the selection of bacteria with antimicrobial resistance reported by Bengtsson-Palme and Larsson (2016), who indicate that concentrations of 0.05, 0.025, and 0.05 µg/L for these antibiotics cannot induce the emergence of resistant strains, respectively [49]. Therefore, our findings indicate an important environmental concern. These high concentrations (from 10^3^ to 10^6^ times higher) can facilitate the selection and persistence of antibiotic-resistant bacteria in aquatic environments and contribute to the dissemination of resistance genes. This is especially problematic in environments where HWW is discharged without prior treatment. This agrees with the studies of Schuster et al. (2022), who detected meropenem at high concentrations in HWW, even above the minimum inhibitory concentration, which is why they concluded that this antibiotic at these concentrations could favor the emergence of resistant strains [50]. Therefore, we speculate that this phenomenon could occur for the concentrations of meropenem identified in this study. From the results presented, the lack of adequate wastewater treatment facilities in our hospital is evident resulting in the uncontrolled release of contaminated HWW into the municipal sewage system, representing a significant risk to public health (Figure 7). This underlines the need for regulations, where the implementation of HWW treatment plants is mandatory to mitigate the risks associated with bacterial pathogens released through these sources. Then again, the issue of antibiotic releases should be addressed through rational antimicrobial stewardship programs, as this could significantly reduce the environmental impact of HWW.

## 5. Conclusions

This study provides evidence of the temporal risks associated with the release of untreated HWW contaminated with β-lactam-resistant pathogens, including the ESKAPE group. Seasonal variation in bacterial loads, the presence of resistance genes, and the release of antibiotics via HWW into the environment contribute to the growing public health concern. Our findings suggest the need for urgent action based on the new regulations on infectious biological waste management and rational use of antimicrobials. In addition, we believe that further research is needed to investigate the impact of HWW on the spread of resistant bacteria to develop more effective strategies to mitigate these risks.

## Figures and Tables

**Table 1:** Analysis of the frequency of β-lactam resistance genes (metallo-β-lactamases and serine β-lactamases) in ESKAPE, emerging, and “other” strains resistant to meropenem, cefepime, and ceftazidime isolated from HWW of the HJM.

Microorganisms	Genes Encoding Carbapenemases *n* (%)							
	Metallo-β-Lactamases			Serine β-Lactamases				Coexistence(*n*/%)
	*bla_IMP_*	*bla_VIM_*	*bla_NDM_*	*bla_OXA-40_*	*bla_OXA-48_*	*bla_OXA-23_*	*bla_KPC_*	
*Escherichia coli* (*n* = 12)	0 (0)	0 (0)	7 (77.3)	0 (0)	4 (30.7)	0 (0)	5 (20.8)	*bla_KPC_ + bla_NDM_* (4/33.3)
*Citrobacter freundii* (*n* = 7)	0 (0)	0 (0)	0 (0)	0 (0)	0 (0)	0 (0)	6 (25)	(0/0)
*Enterobacter* spp. (*n* = 3)	0 (0)	1 (50)	0 (0)	0 (0)	1 (7.7)	0 (0)	2 (8.3)	*bla_VIM_* + *bla_OXA-48_* (1/33.3)
*Acinetobacter baumannii* (*n* = 2)	0 (0)	0 (0)	0 (0)	2 (100)	0 (0)	0 (0)	0 (0)	(0/0)
*Klebsiella pneumoniae* (*n* = 2)	0 (0)	0 (0)	0 (0)	0 (0)	2 (15.4)	0 (0)	0 (0)	(0/0)
*Serratia marcescens* (*n* = 1)	0 (0)	0 (0)	0 (0)	0 (0)	0 (0)	0 (0)	1 (4.1)	(0/0)
*Aeromonas caviae* (*n* = 7)	0 (0)	0 (0)	1 (11.1)	0 (0)	1 (7.7)	0 (0)	7 (29.2)	*bla_KPC_ + bla_NDM_* (1/14.3)
*Aeromonas media* (*n* = 1)	0 (0)	0 (0)	0 (0)	0 (0)	0 (0)	0 (0)	1 (4.1)	(0/0)
*Aeromonas hydrophila* (*n* = 1)	0 (0)	0 (0)	0 (0)	0 (0)	1 (7.7)	0 (0)	0 (0)	(0/0)
*Klebsiella oxytoca* (*n* = 1)	0 (0)	0 (0)	1 (11.1)	0 (0)	0 (0)	0 (0)	1 (4.1)	*bla_KPC_ + bla_NDM_* (1/100)
*Pantoea agglomerans* (*n* = 1)	0 (0)	0 (0)	0 (0)	0 (0)	1 (7.7)	0 (0)	0 (0)	(0/0)
*Pseudomonas* non-*aeruginosa* (*n* = 6)	2 (100)	1 (50)	0 (0)	0 (0)	3 (23.1)	0 (0)	1 (4.1)	*bla_VIM_* + *bla_OXA-48_* (1/16.6)
Total = 44	2 (100)	2 (100)	9 (100)	2 (100)	13 (100)	0 (0)	24 (100)	8 (18.2)

## Data Availability

Bello-López, Juan Manuel (2025), “Microbiological risks to health associated with the release of antibiotic-resistant bacteria through hospital wastewater”, Mendeley Data, V1, https://doi.org/10.17632/mfg4v5ckgk.1 (accessed on 21 April 2025).
